# Incidence and survival of second primary non-Hodgkin lymphoma: A Surveillance, Epidemiology, and End Results-based cohort study

**DOI:** 10.1371/journal.pone.0300330

**Published:** 2024-03-11

**Authors:** Nasha Yu, Weiming Zhang, Xing Zhong, Xiangxiang Song, Wuping Li

**Affiliations:** Departments of Lymphatic and Hematological Oncology, Jiangxi Cancer Hospital (The Second Affiliated Hospital of Nanchang Medical College), Nanchang, Jiangxi, P.R. China; Ruedi Luethy Foundation - Zimbabwe, ZIMBABWE

## Abstract

**Background:**

The aim of this study was to investigate patient survival and factors associated with survival in second primary non-Hodgkin lymphoma (NHL) compared with the first primary NHL.

**Methods:**

The retrospective cohort study used data from the Surveillance, Epidemiology, and End Results (SEER) database between 2000 and 2014. Demographic characteristics, histological types, Ann Arbor stage, and treatment information were collected. Cox proportional hazard models were used to estimate hazard ratios (HRs) and 95% confidence intervals (CIs) for factors associated with overall survival (OS) and cancer-specific survival (CSS) in the first and second primary NHLs.

**Results:**

Of 318,168 cases followed for 5 years, 299,248 patients developed the first primary NHL and 18,920 patients developed the second primary NHL. This study identified a rising incidence of first and second primary NHL from 2000 to 2014. For the second primary NHL, the OS risk was higher when compared to the first primary NHL (HR: 1.13, 95% CI: 1.11 to 1.15, *P* <0.001). Risk factors that negatively affected OS in the first primary NHL included being male, over 40 years of age, certain marital statuses, specific histological types, and advanced disease stages. In contrast, being of White race and having histological types such as Follicular Lymphoma (FL), Marginal Zone Lymphoma (MZL), and mantle B-cell NHL were associated with better OS outcomes. Treatments like surgery, radiation therapy, and chemotherapy were associated with a lower risk of OS and CSS in the first primary NHL. For the second primary NHL, the detrimental risk factors were similar but also included being over the age of 60. Certain histological types showed a lower OS risk relative to diffuse Large B-cell Lymphoma (DLBCL). While surgery and chemotherapy were beneficial for OS, radiation therapy did not improve survival in second primary NHL cases. Notably, undergoing chemotherapy for the first primary cancer increased the OS risk in the second primary NHL, whereas surgery and radiation seemed to offer a protective effect against OS risk in the second primary NHL (all *P* <0.05).

**Conclusion:**

Our findings emphasize the need for tailored strategies in managing the second primary NHL, given the distinct survival patterns and risk factor profiles compared to the first primary NHL. Future research should aim to further elucidate these differences to improve prognosis and treatment approaches for second primary NHL patients.

## Introduction

Non-Hodgkin lymphoma (NHL) is the most common hematological malignancy, accounting for approximately 2.8% of new cancer cases and 2.6% of cancer-related deaths worldwide in 2020 [1–3]. Advances in screening, prevention, diagnosis, and therapeutics have significantly increased the life expectancy and survival of cancer patients, which also increases the risk of developing second primary malignancies (SPMs) [4]. Previous evidence suggests a significant increase in the risk of SPM including NHL after NHL due to improved survival in recent years [5, 6], which has gradually become an important cause of mortality in addition to primary tumors [7]. Thus, understanding the risk of SPM and prognostic survival factors is necessary to guide post-treatment surveillance in cancer survivors.

Previous studies have shown that an increased risk of SPMs after NHL is probably related to a combination of factors including genetic predisposition, molecular background, host immunological status, and therapy administered [8, 9]. In terms of prognosis, previous investigations have shown a higher mortality rate in SPMs than in first primary malignancies [10, 11]. A study by Shen et al. observed a very high mortality rate associated with SPMs, with > 40% of patients dying from their SPM and only 26% of patients dying from their first primary cancer [11]. Thereby, a comparison of the survival between the second and first primary NHL is needed. For prognostic factors for SPMs, family history was found to be associated with survival of second primary cancer in NHL [12]. Research has shown that SPM can develop as a result of the late effects of certain treatments [13]. Treatments for NHL, including chemotherapy and radiotherapy, have been proven to be one of the reasons for the development of SPM [5, 14]. The impact of NHL treatments on the prognosis of second primary NHL also needs to be clarified. The investigation of factors influencing the prognosis of second primary NHL may be helpful for risk stratification of the population and appropriate intervention.

In this population-based study, we aim to analyze compare the survival and factors influencing survival in first primary NHL and second primary NHL. This study may provide a reference for the surveillance and management of second primary NHL.

## Methods

### Data source and study population

This study is a retrospective cohort study. Patients diagnosed with primary NHL between 2000 and 2014 were identified in the Surveillance, Epidemiology, and End Results (SEER) database. The SEER program collects cancer incidence and survival data from population-based cancer registries representing approximately 35% of the U.S. population. We used the “Incidence-SEER Research Plus Data, 17 Registries, Nov 2021 Sub (2000–2019)” database. Included criteria of the study were as follows: (1) patients diagnosed as first or second primary NHL with International Classification of Diseases-Oncology, 3rd edition (ICD-O-3) codes; (2) patients with complete clinicopathological information and survival data; (3) age at diagnosis ≥18 years; (4) patients who were actively followed-up. Excluded criteria were: (1) patients with reported diagnosis source from autopsy or death certificate or with a clinical diagnosis only; (2) patients with two or more primary cancers. Since this study only involves analysis of the publicly available database (SEER) and does not contain any identifying patient information, the ethical approval of this study by the institutional review board is not required. As this is a retrospective study, informed consent of patients was not required.

### Variables collection

Variables from the SEER database included four parts: (1) demographic characteristics: sex (female and male), age at diagnosis (years), race (Black, White, other, and unknown), and marital status at diagnosis [divorced or separated, married or domestic partner, single (never married), widowed, and unknown]; (2) tumor characteristics: histological type [diffuse large B-cell lymphoma (DLBCL), follicular lymphoma (FL), marginal zone lymphoma (MZL), mantle B-cell NHL, NHL, other B-cell, plasma cell neoplasms, T-cell, and unknown] and Ann Arbor stage (stage I, stage II, stage III, stage IV, and unknown); (3) treatment information: surgery, radiotherapy, and chemotherapy; (4) survival information.

### Definition and outcome

Second primary NHL was defined as the primary NHL occurring at least 1 year after the diagnosis of the first primary cancer, and those with the same site and histological subtype as the first primary cancer were excluded [15].

The outcomes of this study included overall survival (OS) and cancer-specific survival (CSS), and factors associated with OS and CSS. OS was defined as the time from diagnosis of primary cancer to death from any cause, and CSS was defined as the time from diagnosis of primary cancer to cancer-related death. Patients were enrolled between 2000 and 2014 and followed up for 5 years.

### Statistical analysis

Continuous variables with normal distribution were expressed as mean +- standard deviation (SD), and the t-test was used to test the difference between the two groups. Data with non-normal distribution were expressed as M (Q_1_, Q_3_), and the difference between groups was analyzed by the Mann-Whitney U rank sum test. Categorical variables were presented as numbers (N) or percentages (%), and the difference between groups was tested by the chi-square test.

To identify potential factors influencing OS and CSS in patients with NHL, univariate and multivariate analyses were performed using the Cox proportional hazards model and Fine-Gray proportional hazards model to determine the potential influencing factors of OS and CSS in NHL patients, respectively. The hazard ratio (HR) and 95% confidence interval (95% CI) were calculated. A two-tailed P-value < 0.05 was considered statistically significant. SAS 9.4 (SAS Institute Inc., Cary, NC, USA) was used for analysis.

## Results

### Basic characteristics of included populations

**Fig 1:**
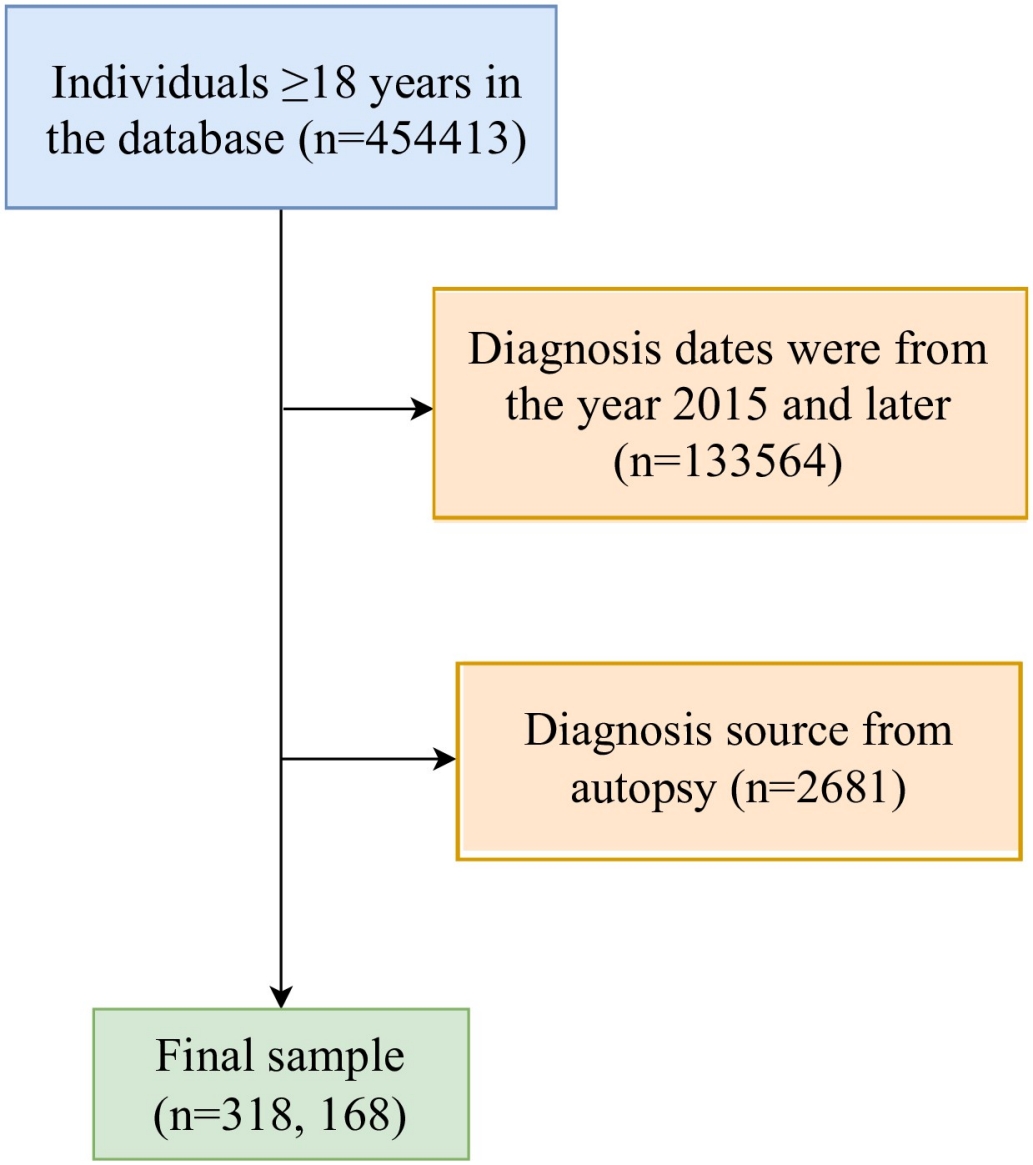
The flowchart displaying the selection procedure of cases in the SEER database. SEER, Surveillance Epidemiology and End Results.

According to the inclusion and exclusion criteria, 318,168 patients were included in this study. Fig 1 illustrates the flowchart outlining the selection process used in this study. Of the total participants, 299,248 patients were diagnosed with their first primary NHL, while 18,920 patients were identified with second primary NHL. The mean age of the patients was 64.89 ± 15.08 years, with 6.0% patients aged <40 years, 29.9% patients aged between 40 to 60 years, and 63.9% patients aged ≥60 years. The predominant racial background of the patients in this study was White, accounting for 83.4% of the total. Regarding marital status at diagnosis, more than half (55.3%) of the patients were married or domestic partners. Regarding the histological type, DLBCL, FL, MZL, mantle B-cell NHL, NHL, other B-cell, plasma cell neoplasms, and T-cell constituted 22.0%, 12.1%, 6.0%, 21.5%, 5.0%, 6.5%, 20.3%, and 6.5%, respectively. The basic characteristics of included populations are shown in Table 1. There were significant differences in sex, age, race, marital status at diagnosis, histological type, Ann Arbor stage, surgery, chemotherapy, and 5-year outcomes between patients with first primary NHL and patients with second primary NHL (all *P* <0.05).

**Table 1:** Basic characteristics of included populations.

Variables	Total (n = 318168)	NHL		Statistics	*P*
		First primary NHL (n = 299248)	Second primary NHL (n = 18920)		
Sex, n (%)				χ^2^ = 661.502	<0.001
Female	140394 (44.13)	133749 (44.70)	6645 (35.12)		
Male	177774 (55.87)	165499 (55.30)	12275 (64.88)		
Age, year, Mean ± SD	64.89 ± 15.08	64.47 ± 15.18	71.54 ± 11.33	t = -81.39	<0.001
Age, year, n (%)				χ^2^ = 3654.468	<0.001
<40	19373 (6.09)	19190 (6.41)	183 (0.97)		
40–60	95223 (29.93)	92400 (30.88)	2823 (14.92)		
≥60	203572 (63.98)	187658 (62.71)	15914 (84.11)		
Race, n (%)				χ^2^ = 267.004	<0.001
Black	29915 (9.40)	28266 (9.45)	1649 (8.72)		
White	265278 (83.38)	248936 (83.19)	16342 (86.37)		
Other	19934 (6.27)	19015 (6.35)	919 (4.86)		
Unknown	3041 (0.96)	3031 (1.01)	10 (0.05)		
Marital status at diagnosis, n (%)				χ^2^ = 508.285	<0.001
Divorced or separated	26718 (8.40)	25307 (8.46)	1411 (7.46)		
Married or domestic partner	176045 (55.33)	164783 (55.07)	11262 (59.52)		
Single (never married)	43942 (13.81)	42307 (14.14)	1635 (8.64)		
Widowed	43919 (13.80)	41052 (13.72)	2867 (15.15)		
Unknown	27544 (8.66)	25799 (8.62)	1745 (9.22)		
Histologic type, n (%)				χ^2^ = 77.010	<0.001
DLBCL	70144 (22.05)	65748 (21.97)	4396 (23.23)		
FL	38480 (12.09)	36356 (12.15)	2124 (11.23)		
MZL	19088 (6.00)	17823 (5.96)	1265 (6.69)		
Mantle B-cell NHL	68450 (21.51)	64448 (21.54)	4002 (21.15)		
NHL	15833 (4.98)	14828 (4.96)	1005 (5.31)		
Other B-cell	20535 (6.45)	19493 (6.51)	1042 (5.51)		
Plasma cell neoplasms	64568 (20.29)	60758 (20.30)	3810 (20.14)		
T-cell	20518 (6.45)	19269 (6.44)	1249 (6.60)		
Unknown	552 (0.17)	525 (0.18)	27 (0.14)		
Ann Arbor stage, n (%)				χ^2^ = 62.913	<0.001
Stage I	48080 (15.11)	44848 (14.99)	3232 (17.08)		
Stage II	27056 (8.50)	25521 (8.53)	1535 (8.11)		
Stage III	28185 (8.86)	26517 (8.86)	1668 (8.82)		
Stage IV	62082 (19.51)	58439 (19.53)	3643 (19.25)		
Unknown	152765 (48.01)	143923 (48.09)	8842 (46.73)		
Surgery, n (%)				χ^2^ = 116.597	<0.001
None/Unknown	127190 (39.98)	118921 (39.74)	8269 (43.71)		
Yes	190978 (60.02)	180327 (60.26)	10651 (56.29)		
Radiation, n (%)				χ^2^ = 0.121	0.727
None/Unknown	2081 (0.65)	1961 (0.66)	120 (0.63)		
Yes	316087 (99.35)	297287 (99.34)	18800 (99.37)		
Chemotherapy, n (%)				χ^2^ = 200.649	<0.001
None/Unknown	152911 (48.06)	142874 (47.74)	10037 (53.05)		
Yes	165257 (51.94)	156374 (52.26)	8883 (46.95)		
Status of 5 years, n (%)				χ^2^ = 715.319	<0.001
Alive	179091 (56.29)	170211 (56.88)	8880 (46.93)		
Dead	139077 (43.71)	129037 (43.12)	10040 (53.07)		
Status of 5 years, n (%)				χ^2^ = 963.967	<0.001
Alive	179091 (56.29)	170211 (56.88)	8880 (46.93)		
Dead-NHL	91624 (28.80)	85669 (28.63)	5955 (31.47)		
Dead-others	47453 (14.91)	43368 (14.49)	4085 (21.59)		
Survival time, months, M (Q_1_, Q_3_)	120.00 (18.00, 120.00)	120.00 (18.00, 120.00)	54.00 (13.00, 120.00)	Z = -24.379	<0.001

Notes: NHL, non-Hodgkin lymphoma; DLBCL, diffuse large B-cell lymphoma; FL, follicular lymphoma; MZL, marginal zone lymphoma; t, t-test; Z, Mann-Whitney U test; χ^2^, Chi-square test; SD, standard deviation; M, Median; Q_1_, 1st Quartile; Q_3_, 3st Quartile.

### Incidences of the first and second primary NHL changed over time

**Fig 2:**
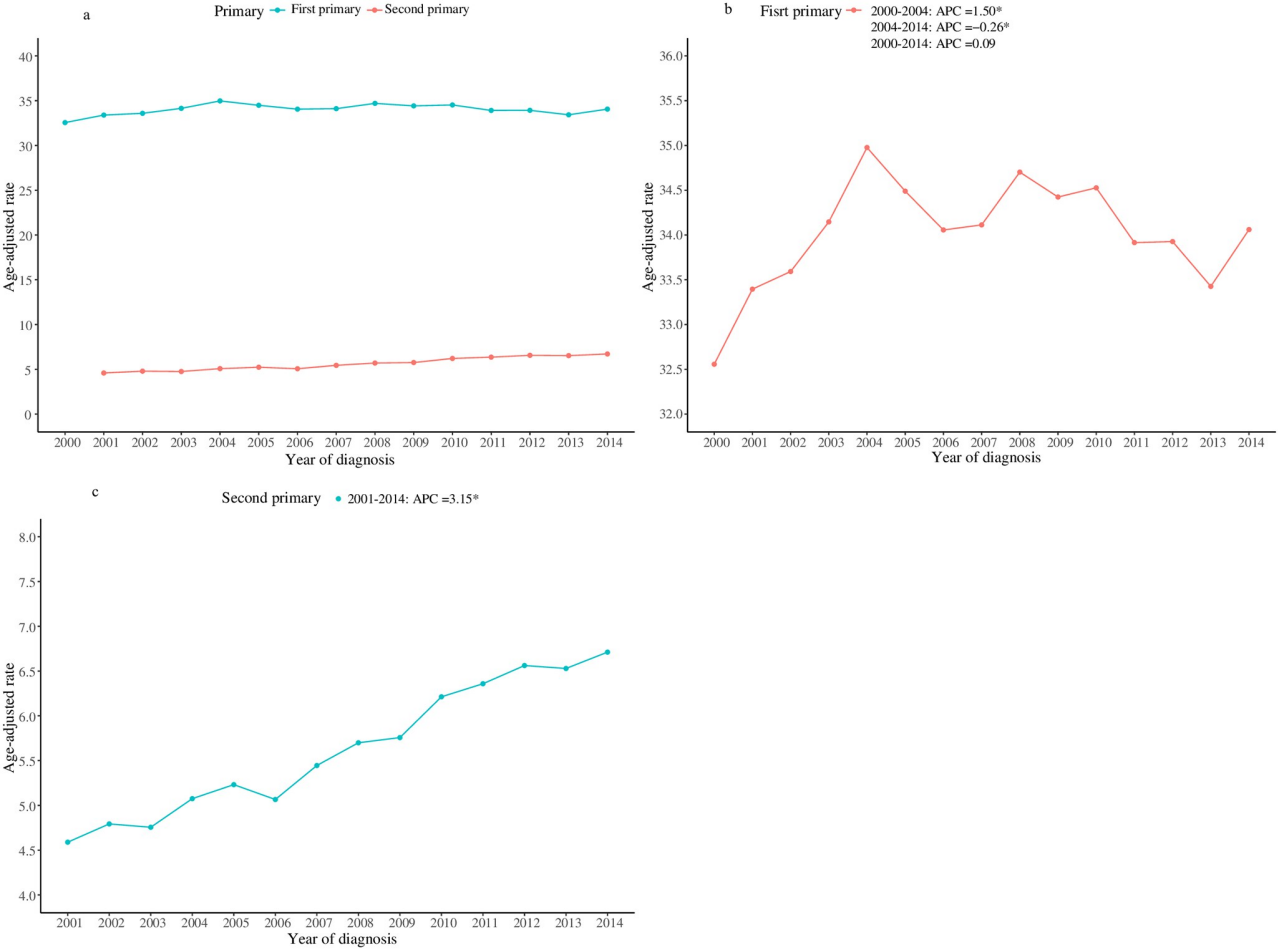
Incidences of the first and second primary NHL changed over time; a, total; b, first primary NHL; c, second primary NHL; NHL, non-Hodgkin lymphoma; APC, annual percent change.

We observed that there was an increase in the incidence of first primary NHL and second primary NHL over time between 2000 and 2014. Incidences of the first and second primary NHL changed over time are depicted in Fig 2.

### Comparison of OS and CSS between the first primary NHL and the second NHL

**Fig 3:**
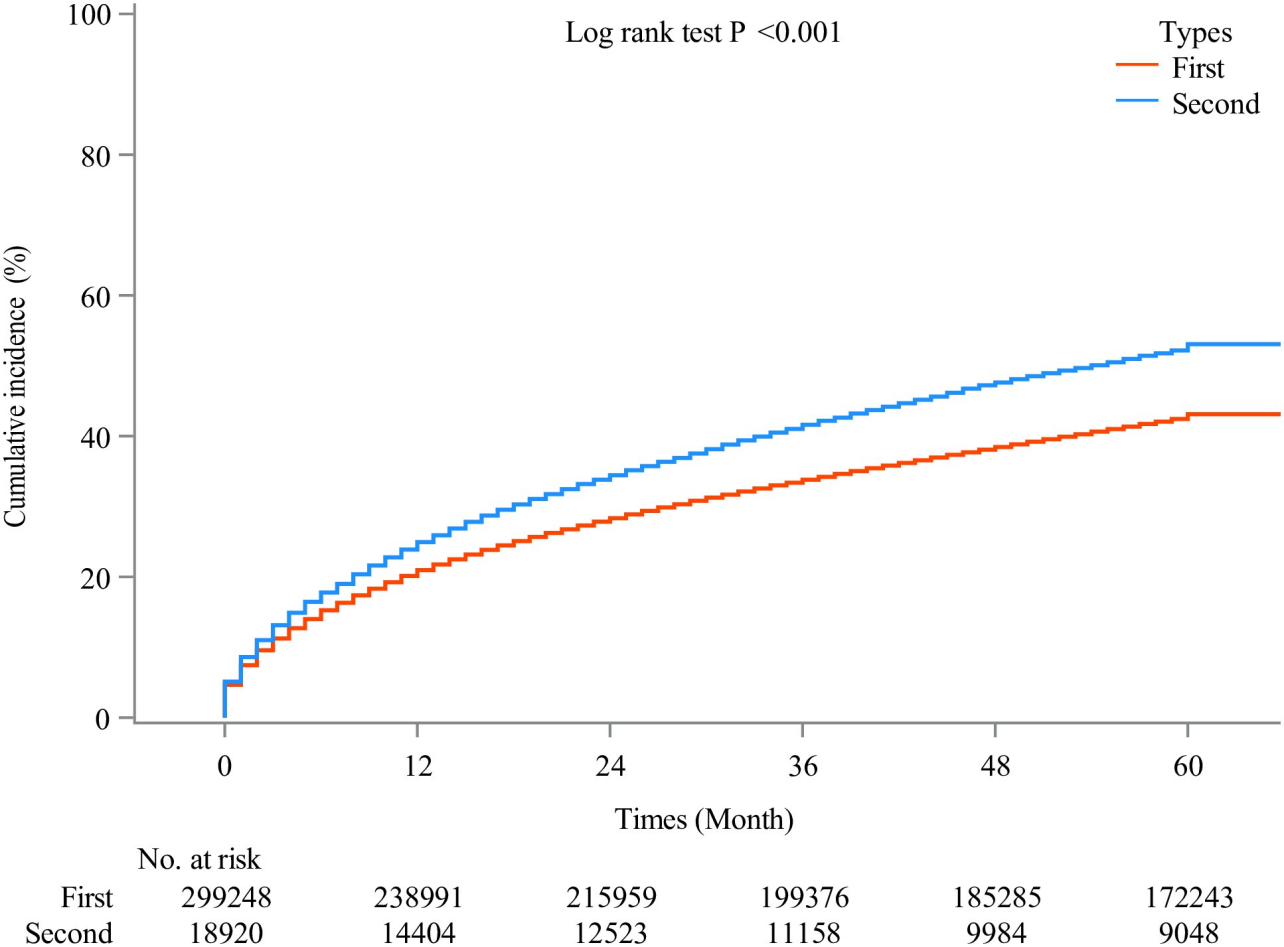
Cumulative incidence of OS in the first and second primary NHL. OS, overall survival; NHL, non-Hodgkin lymphoma.

**Fig 4:**
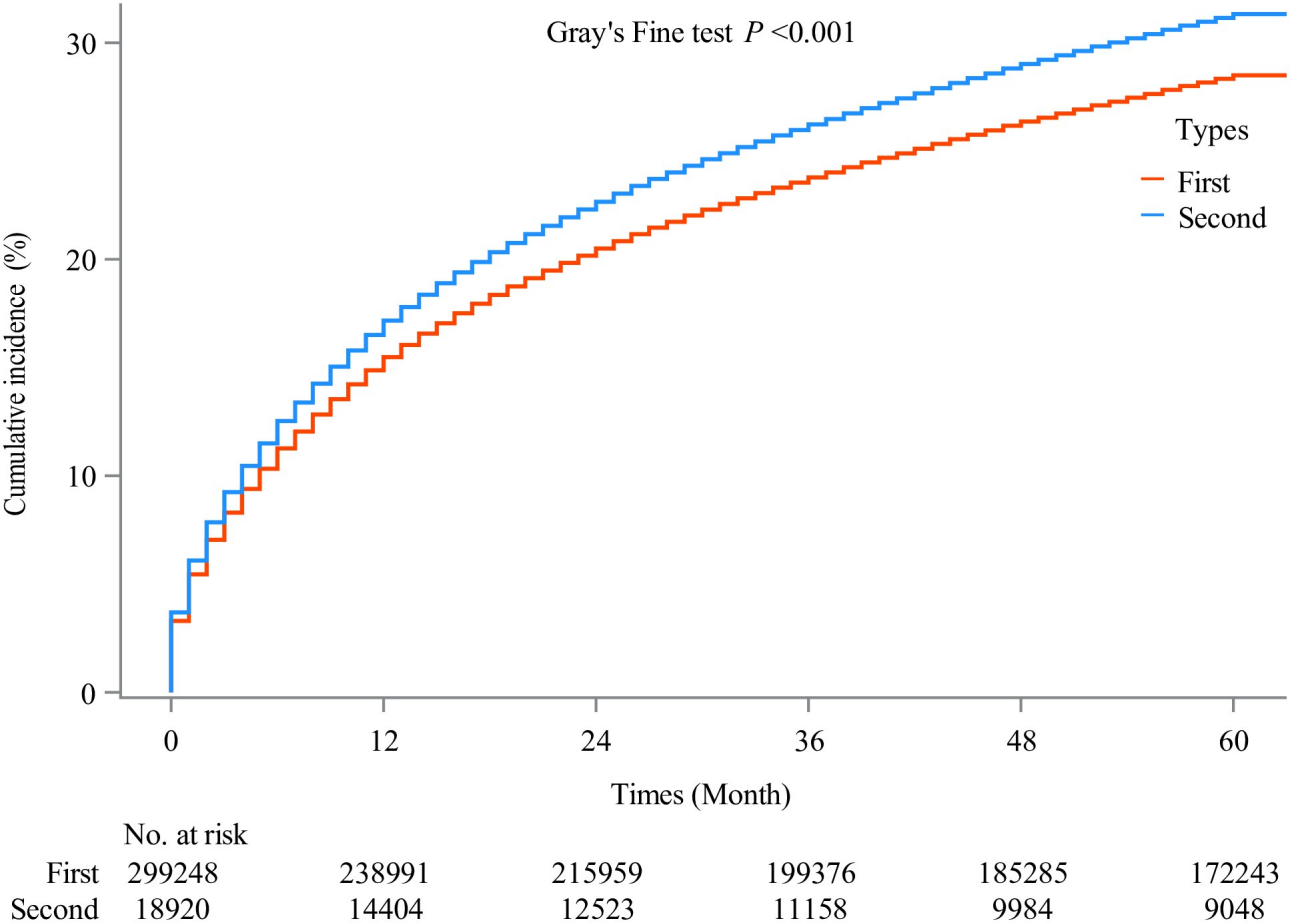
Cumulative incidence of CSS in the first and second primary NHL. CSS, cancer-specific survival; NHL, non-Hodgkin lymphoma.

A higher risk of OS was observed in the second primary NHL (HR: 1.13, 95% CI: 1.11 to 1.15, *P* <0.001), however, there was no difference in CSS between the first primary NHL and the second primary NHL (HR: 1.00, 95% CI: 0.97 to 1.02, *P* = 0.751). A comparison of OS and CSS between the first primary NHL and the second NHL is presented in Table 2. Figures of cumulative incidence of OS and CSS show that the second NHL had a higher cumulative incidence of OS and CSS (Figs 3 and 4).

**Table 2:** Comparison of OS and CSS between the first primary NHL and the second NHL.

Variables	Model1		Model2		Model3	
	HR (95% CI)	*P*	HR (95% CI)	*P*	HR (95% CI)	*P*
OS						
Types						
First	Ref		Ref		Ref	
Second	1.32 (1.29–1.34)	<0.001	1.14 (1.12–1.16)	<0.001	1.13 (1.11–1.16)	<0.001
CSS						
Types						
First	Ref		Ref		Ref	
Second	1.12 (1.09–1.15)	<0.001	1.00 (0.97–1.02)	0.717	1.00 (0.97–1.02)	0.751

Notes: OS, overall survival; CSS, cancer-specific survival; NHL, non-Hodgkin lymphoma; Ref: Reference; HR, hazard ratio; CI, confidence interval; Model 1 adjusted for none; Model 2: adjusted for sex, age, race, marital status at diagnosis, histologic type, and Ann Arbor stage; Model 3 adjusted for sex, age, race, marital status at diagnosis, histologic type, Ann Arbor stage, surgery (not adjusted in OS), and chemotherapy.

### Influencing factors for the OS in the first primary NHL and the second primary NHL

The sex of male (HR: 1.21, 95% CI: 1.20 to 1.23, *P* <0.001), age between 40 and 60 years (HR: 1.13, 95% CI: 1.10 to 1.16, *P* <0.001), age ≥60 years (HR: 2.26, 95% CI: 2.20 to 2.32, *P* <0.001), marital status of divorced or separated (HR: 1.25, 95% CI: 1.22 to 1.27, *P* <0.001), single (never married) (HR: 1.40, 95% CI: 1.37 to 1.42, *P* <0.001), unmarried or domestic partner (HR: 1.27, 95% CI: 1.11 to 1.46, *P* <0.001), and widowed (HR: 1.80, 95% CI: 1.77 to 1.83, *P* <0.001), histological type of other B-cell (HR: 1.11, 95% CI: 1.08 to 1.14, *P* <0.001), plasma cell neoplasms (HR: 1.41, 95% CI: 1.38 to 1.44, *P* <0.001), and T-cell (HR: 1.05, 95% CI: 1.02 to 1.07, *P* <0.001), Ann Arbor stage of II (HR: 1.21, 95% CI: 1.17 to 1.24, *P* <0.001), III (HR: 1.54, 95% CI: 1.50 to 1.58, *P* <0.001), and IV (HR: 1.84, 95% CI: 1.81 to 1.88, *P* <0.001) were related to a higher risk of OS in the first primary NHL. Race of White (HR: 0.91, 95% CI: 0.89 to 0.92, *P* <0.001), histological type of FL (HR: 0.42, 95% CI: 0.41 to 0.43, *P* <0.001), MZL (HR: 0.38, 95% CI: 0.37 to 0.40, *P* <0.001), mantle B-cell NHL (HR: 0.57, 95% CI: 0.56 to 0.59, *P* <0.001), NHL (HR: 0.77, 95% CI: 0.75 to 0.80, *P* <0.001), surgery (yes) (HR: 0.92, 95% CI: 0.90 to 0.93, *P* <0.001), radiation (yes) (HR: 0.83, 95% CI: 0.78 to 0.89, *P* <0.001), and chemotherapy (yes) (HR: 0.88, 95% CI: 0.87 to 0.89, *P* <0.001) were associated with a lower risk of OS in the first primary NHL.

The sex of male (HR: 1.20, 95% CI: 1.15 to 1.26, *P* <0.001), age ≥60 years (HR: 2.06, 95% CI: 1.61 to 2.63, *P* <0.001), race of other (HR: 1.17, 95% CI: 1.05 to 1.30, *P* <0.001), marital status of divorced or separated (HR: 1.15, 95% CI: 1.07 to 1.24, *P* = 0.016), single (never married) (HR: 1.17, 95% CI: 1.09 to 1.26, *P* <0.001), and widowed (HR: 1.59, 95% CI: 1.51 to 1.68, *P* <0.001), histological type of plasma cell neoplasms (HR: 1.16, 95% CI: 1.07 to 1.26, *P* <0.001), Ann Arbor stage of II (HR: 1.10, 95% CI: 1.01 to 1.21, *P =* 0.030), III (HR: 1.36, 95% CI: 1.25 to 1.49, *P* <0.001), and IV (HR: 1.64, 95% CI: 1.53 to 1.76, *P* <0.001), and chemotherapy of the first primary cancer (HR: 1.33, 95% CI: 1.26 to 1.40, *P* <0.001) linked to a higher risk of OS in the second primary NHL. Compared with the histologic type of DLBCL, a lower risk of OS was observed in FL (HR: 0.44, 95% CI: 0.40 to 0.48, *P* <0.001), MZL (HR: 0.41, 95% CI: 0.37 to 0.46, *P* <0.001), mantle B-cell NHL (HR: 0.59, 95% CI: 0.55 to 0.64, *P* <0.001), NHL (0.70, 95% CI: 0.64 to 0.77, *P* <0.001), and other B-cell (HR: 0.85, 95% CI: 0.77 to 0.94, *P* <0.001). Surgery (yes) (HR: 0.89, 95% CI: 0.85 to 0.94, *P* <0.001), chemotherapy (yes) (HR: 0.87, 95% CI: 0.84 to 0.91, *P* <0.001), treatment of the first primary cancer of surgery (HR: 0.88, 95% CI: 0.85 to 0.92, *P* <0.001), and radiation (HR: 0.79, 95% CI: 0.67 to 0.93, *P* = 0.006) were related to a lower risk of OS in the second primary NHL. Table 3 shows the influencing factors for the OS in the first and second primary NHLs.

**Table 3:** Influencing factors for the OS in the first and second primary NHLs.

Variables	First primary NHL		Second primary NHL	
	HR (95% CI)	*P*	HR (95% CI)	*P*
Sex				
Female	Ref		Ref	
Male	1.21 (1.20–1.23)	<0.001	1.20 (1.15–1.26)	<0.001
Age, year				
<40	Ref		Ref	
40–60	1.13 (1.10–1.16)	<0.001	1.12 (0.87–1.43)	0.394
≥60	2.26 (2.20–2.32)	<0.001	2.06 (1.61–2.63)	<0.001
Race				
Black	Ref		Ref	
White	0.91 (0.89–0.92)	<0.001	1.01 (0.94–1.08)	0.797
Other	0.98 (0.96–1.01)	0.239	1.17 (1.05–1.30)	0.005
Unknown	0.65 (0.61–0.70)	<0.001	-	-
Marital status at diagnosis				
Married or Domestic Partner	Ref		Ref	
Divorced or Separated	1.25 (1.22–1.27)	<0.001	1.15 (1.07–1.24)	<0.001
Single (never married)	1.40 (1.37–1.42)	<0.001	1.17 (1.09–1.26)	<0.001
Widowed	1.80 (1.77–1.83)	<0.001	1.59 (1.51–1.68)	<0.001
Unknown	0.95 (0.93–0.97)	<0.001	0.88 (0.81–0.95)	<0.001
Histologic Type				
DLBCL	Ref		Ref	
FL	0.42 (0.41–0.43)	<0.001	0.44 (0.40–0.48)	<0.001
MZL	0.38 (0.37–0.40)	<0.001	0.41 (0.37–0.46)	<0.001
Mantle B-cell NHL	0.57 (0.56–0.59)	<0.001	0.59 (0.55–0.64)	<0.001
NHL	0.77 (0.75–0.80)	<0.001	0.70 (0.64–0.77)	<0.001
Other B-cell	1.11 (1.08–1.14)	<0.001	0.85 (0.77–0.94)	0.001
Plasma cell neoplasms	1.41 (1.38–1.44)	<0.001	1.16 (1.07–1.26)	<0.001
T-cell	1.05 (1.02–1.07)	<0.001	0.93 (0.85–1.01)	0.100
Unknown	1.48 (1.33–1.66)	<0.001	1.70 (1.09–2.64)	0.019
Ann Arbor stage				
Stage I	Ref		Ref	
Stage II	1.21 (1.17–1.24)	<0.001	1.10 (1.01–1.21)	0.030
Stage III	1.54 (1.50–1.58)	<0.001	1.36 (1.25–1.49)	<0.001
Stage IV	1.84 (1.81–1.88)	<0.001	1.64 (1.53–1.76)	<0.001
Unknown	1.18 (1.15–1.21)	<0.001	1.10 (1.02–1.19)	0.012
Surgery				
None/Unknown	Ref		Ref	
Yes	0.92 (0.90–0.93)	<0.001	0.89 (0.85–0.94)	<0.001
Radiation				
None/Unknown	Ref		Ref	
Yes	0.83 (0.78–0.89)	<0.001	0.97 (0.76–1.23)	0.776
Chemotherapy				
None/Unknown	Ref		Ref	
Yes	0.88 (0.87–0.89)	<0.001	0.87 (0.84–0.91)	<0.001
Treatment of the first primary cancer				
Surgery				
None/Unknown			Ref	
Yes			0.88 (0.85–0.92)	<0.001
Radiation				
None/Unknown			Ref	
Yes			0.79 (0.67–0.93)	0.006
Chemotherapy				
None/Unknown			Ref	
Yes			1.33 (1.26–1.40)	<0.001

Notes: OS, overall survival; NHL, non-Hodgkin lymphoma; DLBCL, diffuse large B-cell lymphoma; FL, follicular lymphoma; MZL, marginal zone lymphoma; Ref: Reference, HR: hazard ratio, CI: confidence interval.

### Influencing factors for the CSS in the first primary NHL and the second primary NHL

The sex of male was associated with a high risk of CSS in the first primary NHL (HR: 1.12, 95% CI: 1.11 to 1.14, *P* <0.001) and the second primary NHL (HR: 1.16, 95% CI: 1.09 to 1.23, *P* <0.001). Age between 40 to 60 years (HR: 1.20, 95% CI: 1.16 to 1.25, *P* <0.001) and age ≥60 years (HR: 2.09, 95% CI: 2.02 to 2.16, *P* <0.001) were associated with the risk of CSS in the first primary NHL, however, only age ≥60 years was related to a higher risk of CSS in the second primary NHL (HR: 1.67, 95% CI: 1.25 to 2.24, *P* <0.001). Race of White was found to be associated with a decreased risk of CSS in the first primary NHL (HR: 0.93, 95% CI: 0.91 to 0.95, *P* <0.001). Marital status of divorced or separated (HR: 1.18, 95% CI: 1.15 to 1.21, *P* <0.001), and single (never married) (HR: 1.30, 95% CI: 1.28 to 1.33, *P* <0.001) were related to a higher risk of CSS in the first primary NHL. The marital status of the widowed was related to a higher risk of CSS both in the first primary NHL (HR: 1.58, 95% CI: 1.55 to 1.61, *P* <0.001) and the second primary NHL (HR: 1.44, 95% CI: 1.34 to 1.55, *P* <0.001).

Compared with DLBCL, FL, MZL, mantle B-cell NHL, and NHL were associated with a lower risk of CSS in the first primary NHL and the second primary NHL (all *P* <0.001). Other B-cell was related to the risk of CSS in the first primary NHL (HR: 1.23, 95% CI: 1.20 to 1.27, *P* <0.001). Compared with Ann Arbor stage I, Ann Arbor stage II was associated with CSS in the first primary NHL (HR: 1.28, 95% CI: 1.24 to 1.33, *P* <0.001), stage III, and stage IV were related to a higher risk of CSS in the first primary NHL and the second primary NHL (all *P* <0.05). Surgery was related to a lower risk of CSS in both the first primary NHL (HR: 0.92, 95% CI: 0.91 to 0.94, *P* <0.001) and the second primary NHL (HR: 0.87, 95% CI: 0.82 to 0.93, *P* <0.001). Radiation was associated with CSS in the first primary NHL (HR: 0.81, 95% CI: 0.74 to 0.87, *P* <0.001), however, was not associated with CSS in the second primary NHL (HR: 0.93, 95% CI: 0.68 to 1.29, *P* = 0.678). Chemotherapy was related to a higher risk of CSS in both the first primary NHL (HR: 1.09, 95% CI: 1.08 to 1.11, *P* <0.001) and the second primary NHL (HR: 1.15, 95% CI: 1.09 to 1.23, *P* <0.001). Regarding the treatment of the first primary cancer, surgery (HR: 0.93, 95% CI: 0.88 to 0.98, *P* = 0.011) was associated with a lower risk of CSS of second primary NHL, however, chemotherapy (HR: 1.37, 95% CI: 1.28 to 1.46, *P* <0.001) were related to a higher risk of CSS in the second primary NHL. Influencing factors for the CSS in the first primary NHL and the second primary NHL are shown in Table 4.

**Table 4:** Influencing factors for the CSS in the first primary NHL and the second primary NHL.

Variables	First primary NHL		Second primary NHL	
	HR (95% CI)	*P*	HR (95% CI)	*P*
Sex				
Female	Ref		Ref	
Male	1.12 (1.11–1.14)	<0.001	1.16 (1.09–1.23)	<0.001
Age, year				
<40	Ref		Ref	
40–60	1.20 (1.16–1.25)	<0.001	1.08 (0.80–1.46)	0.620
60+	2.09 (2.02–2.16)	<0.001	1.67 (1.25–2.24)	<0.001
Race				
Black	Ref		Ref	
White	0.93 (0.91–0.95)	<0.001	1.06 (0.97–1.16)	0.181
Other	0.97 (0.94–1.01)	0.099	1.08 (0.94–1.25)	0.264
Unknown	0.17 (0.15–0.21)	<0.001	-	<0.001
Marital status at diagnosis				
Married or Domestic Partner	Ref		Ref	
Divorced or Separated	1.18 (1.15–1.21)	<0.001	1.05 (0.95–1.16)	0.333
Single (never married)	1.30 (1.28–1.33)	<0.001	1.07 (0.97–1.18)	0.155
Widowed	1.58 (1.55–1.61)	<0.001	1.44 (1.34–1.55)	<0.001
Unknown	0.81 (0.79–0.84)	<0.001	0.77 (0.69–0.85)	<0.001
Histologic Type				
DLBCL	Ref		Ref	
FL	0.37 (0.36–0.38)	<0.001	0.37 (0.33–0.42)	<0.001
MZL	0.26 (0.25–0.27)	<0.001	0.26 (0.22–0.31)	<0.001
Mantle B-cell NHL	0.51 (0.49–0.52)	<0.001	0.50 (0.45–0.55)	<0.001
NHL	0.71 (0.69–0.74)	<0.001	0.60 (0.53–0.68)	<0.001
Other B-cell	1.23 (1.20–1.27)	<0.001	0.95 (0.84–1.07)	0.373
Plasma cell neoplasms	1.56 (1.51–1.61)	<0.001	1.16 (1.04–1.29)	0.008
T-cell	1.17 (1.13–1.20)	<0.001	1.10 (0.99–1.23)	0.081
Unknown	1.65 (1.45–1.86)	<0.001	1.94 (1.12–3.34)	0.017
Ann Arbor stage				
Stage I	Ref		Ref	
Stage II	1.28 (1.24–1.33)	<0.001	1.09 (0.97–1.23)	0.165
Stage III	1.73 (1.68–1.79)	<0.001	1.43 (1.28–1.59)	<0.001
Stage IV	2.16 (2.11–2.22)	<0.001	1.71 (1.56–1.88)	<0.001
Unknown	1.16 (1.13–1.20)	<0.001	1.09 (0.98–1.21)	0.111
Surgery				
None/Unknown	Ref		Ref	
Yes	0.92 (0.91–0.94)	<0.001	0.87 (0.82–0.93)	<0.001
Radiation				
None/Unknown	Ref		Ref	
Yes	0.81 (0.74–0.87)	<0.001	0.93 (0.68–1.29)	0.678
Chemotherapy				
None/Unknown	Ref		Ref	
Yes	1.09 (1.08–1.11)	<0.001	1.15 (1.09–1.23)	<0.001
Treatment of the first primary cancer				
Surgery				
None/Unknown			Ref	
Yes			0.93 (0.88–0.98)	0.011
Radiation				
None/Unknown			Ref	
Yes			0.88 (0.71–1.10)	0.277
Chemotherapy				
None/Unknown			Ref	
Yes			1.37 (1.28–1.46)	<0.001

Notes: CSS, cancer-specific survival; NHL, non-Hodgkin lymphoma; DLBCL, diffuse large B-cell lymphoma; FL, follicular lymphoma; MZL, marginal zone lymphoma; Ref: Reference, HR: hazard ratio, CI: confidence interval.

## Discussion

In this study, the incidence of the second primary NHL increased over time between 2000 and 2014. In terms of prognosis, a higher risk of OS was observed in the second primary NHL compared with OS in the first primary NHL. Male gender, age of 40 years or older, being divorced or separated, single, or in an unmarried or domestic partner, having a histological type of other B-cell, plasma cell neoplasms, or T-cell, and advanced Ann Arbor stages were associated with an increased risk of OS in first primary NHL patients. Conversely, factors associated with a decreased risk of OS in these first primary NHL patients included being of White race, having histological types such as FL, MZL, mantle B-cell NHL, undergoing surgery, radiation therapy, and receiving chemotherapy. Being male, aged 60 years or older, having a marital status of divorced or separated, single (never married), or widowed, having plasma cell neoplasms, and having higher Ann Arbor stages were related to an increased risk of OS in the second primary NHL. In contrast, when compared to the histological type of DLBCL, lower OS risks were observed in FL, MZL, mantle B-cell NHL, other B-cell NHL, and NHL overall. Surgery and chemotherapy treatments were linked to the risk of OS in second primary NHL. However, radiation treatment did not benefit survival in in second primary NHL. Additionally, undergoing chemotherapy for the first primary cancer was linked to a higher OS risk in the second primary NHL. Nevertheless, surgery and radiation as treatments for the first primary cancer were associated with a lower risk of OS in the second primary NHL.

We observed a significant increase in the incidence of first and second primary NHL in patients between 2000 and 2014. There is an apparent increase in the incidence of NHL in recent years, which is likely related to the overall improvement and availability of newer diagnostic technology, improvements in the treatment paradigm, and follow-up after remission, all of which have contributed to improved overall survival over time [16]. In a 2011 meta-analysis, Pirani et al found pooled relative risks of 1.88 for any SPM among patients with NHL [17]. In a recent study, the author evaluated the trends of SPM risk among NHL survivors over the past four decades and found that the relative and cumulative risk for both hematological and solid second cancers after NHL increased over time, with the increasing trend being more pronounced for hematological malignancies compared with solid tumors [5]. The highest standardized incidence ratio was observed in the period 2010–2016, and the lowest in the period 1975–1989 [5]. A study examining the incidence and epidemiology of NHL and the risk of SPMs in 22,466 survivors in Israel with 30 years of follow-up observed an increased incidence of SPMs, both solid tumors and hematological malignancies [16]. Several factors are likely to account for this observation. Changes in the treatment of NHL patients in recent years, including an increase in the proportion of patients receiving intensive chemotherapy and hematopoietic stem cell transplantation (HSCT), maybe the main reason for the increased risk of SPMs in NHL patients after surgery [5]. Studies reporting the risk of second primary NHL in different study periods would be helpful.

In this study, the second primary NHL had a worse OS than the first primary NHL. A previous study demonstrated that patients with a SPM have higher mortality rates than those with a single tumor [15]. Liou et al. found that patients with well-differentiated thyroid carcinoma and metachronous SPM had a worse prognosis than patients without SPM [18]. A propensity score analysis indicated that compared with mortalities from prior cancers, more cancer-related mortalities were observed in patients with pancreatic ductal adenocarcinomas as a second primary tumor [19]. Chattopadhyay et al. found that NHL survivors who developed a second cancer were far less likely to survive than those who did not [12]. Thus, it is of great importance to pay attention to the survival factors of SPM for disease control and improvement of survival.

We found patients’ basic characteristics including sex, age, marital status, and race were influencing factor of OS in both first primary and second primary NHL. Male gender was related to a higher risk of OS in both first primary and second primary NHL. Age of 40 years or older was associated with an increased risk of OS in the first primary NHL while aged 60 years or older was linked to increased risk of OS in the second primary NHL. Marital status of divorced or separated, single (never married) or widowed were associated with an increased risk of OS in the first and second primary NHL. Being of White race was related to a decreased risk of OS in these first primary NHL patients, however, was not associated with OS in the second primary NHL patients. According to a previous study, age, race, and marital status showed a significant association with OS and CSS in primary gastric DLBCL patients [20]. A registry-based clinical cohort study investigating the association between socioeconomic position and prognostic markers in 6,234 individuals enrolled in a national clinical database in Denmark reported that living with a partner and being female were associated with reduced risk of being diagnosed with advanced disease of NHL [21]. A study by Frederiksen et al. reported that mortality was increased in NHL patients who were singles [22]. Better financial and psychological support may be beneficial for treatments, so married patients were associated with better prognosis [20]. Race has been found to be an important factor in both management decisions, as well as in survivorship. In a cohort of 1,605 patients with Hodgkin lymphoma treated in the Children’s Oncology Group trials between 2002 and 2012,69 non-White patients (pooled Black and Hispanic) had a 1.88 -fold increased risk of mortality in multivariable analyses [23]. Abodunrin et al. found significant differences in the disease distribution and relative survival of NHL patients among the different racial groups in the United States [24]. Li et al. found a significantly higher five-year relative survival in Whites with stages I-IV DLBCL compared to Blacks, Asians, and Pacific Islanders, while Blacks have the worst five-year relative survival among patients with stage I-III, and Asians/Pacific Islanders have the worst survival in stage IV [25].

NHL represents a heterogeneous group of diseases, for which the biological behavior, treatment, clinical course, thus, survival vary according to the subtype [26]. In the case of second primary NHL, lower OS risks were noted in histological types such as FL, MZL, mantle B-cell NHL, other B-cell NHLs, in comparison to DLBCL. Conversely, plasma cell neoplasms were associated with an increased risk of OS in both first and second primary NHL cases. The evaluation of 5-year net survival according to subtype showed the highest survival for FL (70%) and the lowest survival for DLBCL (47%) [27]. Another analysis of cumulative survival rates from diagnosis for NHL subtypes found that 5-year survival rates were highest for patients with mucosa-associated lymphoid tissue lymphoma (90.8%), followed by FL (87.6%), DLBCL (69.0%), and mantle-cell lymphoma (57.1%) [28]. We also found that other B-cell and T-cell were associated with a higher risk of OS in the primary first NHL, however, associated with a decreased risk of OS in the primary second NHL. Further studies are necessary to detect associations between specific NHL types and OS or CSS in the first primary and the second primary NHL. Advanced Ann Arbor stages were found to be associated with OS and CSS in the first primary NHL and second primary NHL. Ann Arbor stages were observed to be associated with OS and CSS in primary gastric DLBCL patients [20]. Among these independent prognostic factors, Ann Arbor stage is associated with prognosis in many cases of primary NHLs [29]. The current five-year survival of NHL for stage I disease at diagnosis is 83.5% while the survival for stage IV disease is 63.3% [2]. The cancer stage disparity in survival outcomes among patients with first and second primary NHL persisted.

With respect to treatment, surgery, radiation, and chemotherapy were found to be associated with a lower risk of OS and CSS in the first primary NHL. A study by Liu et al. suggested that chemotherapy and surgery are beneficial to patients with primary small intestinal DLBCL [30]. Chemotherapy and radiation therapy showed a significant association with OS and CSS in primary gastric DLBCL patients [20]. Another previous study suggested that early DLBCL without radiation therapy has increased cardiac mortality compared with those with radiation therapy [31]. However, radiation therapy was not associated with improved OS and CSS in the second primary NHL. In a previous study, radiation therapy did not increase mortality from SPMs in adolescent and young adult patients with lymphoid malignancies [32]. Several side effects threatening the normal tissues of patients after treatment of radiation therapy may be the reason why radiation therapy was not beneficial to the second primary NHL [33]. Modern radiation therapy for SPMs in warrants further evaluation in randomized trials. Treatments for the first primary cancer also affect the risk for subsequent cancers [5]. We found that treatment for the first primary NHL with surgery and chemotherapy was related to OS and CSS in the second primary NHL. In head and neck cancer patients, prior treatment of primary cancer often affects the treatment of esophageal SPMs [34]. Given antitumor therapy is considered to affect the survival of SPMs, proper antitumor therapy must be suggested.

Our study offers significant clinical implications. Identifying specific risk factors associated with survival rates in second primary NHL allows clinicians to recognize high-risk patients early and implement more intensive monitoring and assessment. the differences observed in treatment responses between first and second primary NHL suggest that more personalized treatment strategies may be necessary for second primary NHL. The study’s findings, which indicate differing efficacies of treatments such as surgery and chemotherapy in second primary NHL compared to first primary NHL, can help guide clinicians in making more targeted treatment choices. Understanding that patients with first primary NHL who have undergone chemotherapy may face higher survival risks when developing second primary NHL suggests that the impact of prior treatments should be considered in subsequent care. The results of the study can serve as a basis for future research to further explore how to improve treatment outcomes and survival rates for second primary NHL patients.

Based on the SEER database, the sample size of this study is large and representative. To the best of our knowledge, this study was first to analyze the prevalence and survival factors of the second primary NHL, which provides a reference for the selection and monitoring of second primary NHL by comparison with first primary NHL. However, several limitations need to be noticed. Firstly, our study is subject to the inherent limitations of retrospective registry-based data. In contrast to a prospective study, the observational nature of registry data allows for significant biases related to imbalance of demographic and clinical factors between the groups of interest and missing covariates that could affect the inferred effects of other covariates on survival outcomes. Secondly, treatment-specific information such as radiation dose and potential confounders during follow-up were not taken into account. Thirdly, recurrence and metastasis of the first primary NHL can be considered as a subsequent SPM, but the definition of SPM in this study excluded patients with exactly the same tumor site and histological subtype. Although we have set a minimum one-year interval between the diagnoses of the first and second cancers, a one-year interval may be considered relatively short to conclusively distinguish between primary and subsequent cancers. Future research may benefit from exploring longer intervals to potentially yield more definitive conclusions about second primary cancers. Fourthly, information on the stage of patients and specific treatments was unavailable for a significant proportion of the cohort. This limitation underscores the need for a cautious interpretation of the influence of these variables on survival outcomes. Future research should aim to address these data gaps to better understand the complex interplay of treatments and outcomes in NHL survivorship.

## Conclusion

The incidence of the second primary NHL has risen from 2000 to 2014. The prognosis for the second primary NHL showed a higher OS risk compared to the first primary NHL. Identified risk factors for OS in the first primary NHL patients included male gender, age over 40, and certain marital statuses, as well as specific histological types and advanced stages of the disease. Conversely, White race and histological types such as FL, MZL, and mantle B-cell NHL, along with surgery, radiation therapy, and chemotherapy, were linked to improved OS. In the second primary NHL, similar risk factors were detrimental, with the addition of age over 60. However, certain histological types demonstrated a lower OS risk compared to DLBCL, and while surgery and chemotherapy influenced OS, radiation therapy did not show a survival benefit for second primary NHL. Importantly, chemotherapy for the first primary cancer was associated with increased OS risk in the second primary NHL, whereas surgery and radiation appeared to reduce OS risk.

## Supporting information

S1 Data (RAR)

## References

[1] Sung H, Ferlay J, Siegel RL, Laversanne M, Soerjomataram I, Jemal A, et al. Global Cancer Statistics 2020: GLOBOCAN Estimates of Incidence and Mortality Worldwide for 36 Cancers in 185 Countries. CA: a cancer journal for clinicians. 2021;71(3):209–49. Epub 2021/02/05. doi: 10.3322/caac.21660 . 33538338

[2] Thandra KC, Barsouk A, Saginala K, Padala SA, Barsouk A, Rawla P. Epidemiology of Non-Hodgkin’s Lymphoma. Medical sciences (Basel, Switzerland). 2021;9(1). Epub 2021/02/13. doi: 10.3390/medsci9010005 . 33573146 PMC7930980

[3] Mafra A, Laversanne M, Gospodarowicz M, Klinger P, De Paula Silva N, Piñeros M, et al. Global patterns of non-Hodgkin lymphoma in 2020. International journal of cancer. 2022;151(9):1474–81. Epub 2022/06/14. doi: 10.1002/ijc.34163 . 35695282

[4] Alhamadh MS, Alanazi RB, Algarni ST, Alhuntushi AAR, Alshehri MQ, Chachar YS, et al. A Descriptive Study of the Types and Survival Patterns of Saudi Patients with Multiple Primary Solid Malignancies: A 30-Year Tertiary Care Center Experience. Current oncology (Toronto, Ont). 2022;29(7):4941–55. Epub 2022/07/26. doi: 10.3390/curroncol29070393 . 35877253 PMC9315520

[5] Li J, Peng F, Huang H, Cai Z. Trends in the risk of second primary malignances after non-Hodgkin’s lymphoma. American journal of cancer research. 2022;12(6):2863–75. Epub 2022/07/12. . 35812045 PMC9251676

[6] Joelsson J, Wästerlid T, Rosenquist R, Jakobsen LH, El-Galaly TC, Smedby KE, et al. Incidence and time trends of second primary malignancies after non-Hodgkin lymphoma: a Swedish population-based study. Blood advances. 2022;6(8):2657–66. Epub 2022/01/19. doi: 10.1182/bloodadvances.2021006369 . 35042239 PMC9043935

[7] Liu W, Ji X, Song Y, Wang X, Zheng W, Lin N, et al. Improving survival of 3760 patients with lymphoma: Experience of an academic center over two decades. Cancer medicine. 2020;9(11):3765–74. Epub 2020/04/14. doi: 10.1002/cam4.3037 . 32281275 PMC7286476

[8] Goyal A, O’Leary D, Goyal K, Rubin N, Bohjanen K, Hordinsky M, et al. Increased risk of second primary hematologic and solid malignancies in patients with mycosis fungoides: A Surveillance, Epidemiology, and End Results analysis. Journal of the American Academy of Dermatology. 2020;83(2):404–11. Epub 2019/08/03. doi: 10.1016/j.jaad.2019.07.075 . 31374302 PMC7021276

[9] Pasqual E, Schonfeld S, Morton LM, Villoing D, Lee C, Berrington de Gonzalez A, et al. Association Between Radioactive Iodine Treatment for Pediatric and Young Adulthood Differentiated Thyroid Cancer and Risk of Second Primary Malignancies. Journal of clinical oncology: official journal of the American Society of Clinical Oncology. 2022;40(13):1439–49. Epub 2022/01/20. doi: 10.1200/JCO.21.01841 . 35044839 PMC9061144

[10] Dinnessen MAW, Visser O, Tonino SH, van der Poel MWM, Blijlevens NMA, Kersten MJ, et al. The impact of prior malignancies on the development of second malignancies and survival in follicular lymphoma: A population-based study. EJHaem. 2020;1(2):489–97. Epub 2020/10/08. doi: 10.1002/jha2.108 . 35844986 PMC9175939

[11] Shen C, Wang C, He T, Cai Z, Yin X, Yin Y, et al. Long-term survival among patients with gastrointestinal stromal tumors diagnosed after another malignancy: a SEER population-based study. World journal of surgical oncology. 2020;18(1):88. Epub 2020/05/08. doi: 10.1186/s12957-020-01868-x . 32375797 PMC7204066

[12] Chattopadhyay S, Zheng G, Sud A, Sundquist K, Sundquist J, Försti A, et al. Second primary cancers in non-Hodgkin lymphoma: Family history and survival. International journal of cancer. 2020;146(4):970–6. Epub 2019/05/06. doi: 10.1002/ijc.32391 . 31054153

[13] Mazzone E, Mistretta FA, Knipper S, Palumbo C, Tian Z, Pecoraro A, et al. Long-term incidence of secondary bladder and rectal cancer in patients treated with brachytherapy for localized prostate cancer: a large-scale population-based analysis. BJU international. 2019;124(6):1006–13. Epub 2019/05/31. doi: 10.1111/bju.14841 . 31144770

[14] Turcotte LM, Liu Q, Yasui Y, Henderson TO, Gibson TM, Leisenring W, et al. Chemotherapy and Risk of Subsequent Malignant Neoplasms in the Childhood Cancer Survivor Study Cohort. Journal of clinical oncology: official journal of the American Society of Clinical Oncology. 2019;37(34):3310–9. Epub 2019/10/18. doi: 10.1200/JCO.19.00129 . 31622130 PMC7001784

[15] Donin N, Filson C, Drakaki A, Tan HJ, Castillo A, Kwan L, et al. Risk of second primary malignancies among cancer survivors in the United States, 1992 through 2008. Cancer. 2016;122(19):3075–86. Epub 2016/07/06. doi: 10.1002/cncr.30164 . 27377470 PMC6192520

[16] Tadmor T, Liphshitz I, Silverman B, Polliack A. Incidence and epidemiology of non-Hodgkin lymphoma and risk of second malignancy among 22 466 survivors in Israel with 30 years of follow-up. Hematological oncology. 2017;35(4):599–607. Epub 2016/05/31. doi: 10.1002/hon.2302 . 27238496

[17] Pirani M, Marcheselli R, Marcheselli L, Bari A, Federico M, Sacchi S. Risk for second malignancies in non-Hodgkin’s lymphoma survivors: a meta-analysis. Annals of oncology: official journal of the European Society for Medical Oncology. 2011;22(8):1845–58. Epub 2011/02/12. doi: 10.1093/annonc/mdq697 . 21310758

[18] Liou MJ, Tsang NM, Hsueh C, Chao TC, Lin JD. Therapeutic Outcome of Second Primary Malignancies in Patients with Well-Differentiated Thyroid Cancer. International journal of endocrinology. 2016;2016:9570171. Epub 2016/04/28. doi: 10.1155/2016/9570171 . 27118971 PMC4828550

[19] He C, Zhang Y, Cai Z, Lin X. Effect of prior cancer on survival outcomes for patients with pancreatic adenocarcinoma: a propensity score analysis. BMC cancer. 2019;19(1):509. Epub 2019/05/31. doi: 10.1186/s12885-019-5744-8 . 31142278 PMC6542019

[20] Lu G, Lin Z, Ruan Y, Huang H, Lin J, Pan J. A Novel Prognostic Model for Patients with Primary Gastric Diffuse Large B-Cell Lymphoma. Journal of oncology. 2022;2022:9636790. Epub 2022/11/08. doi: 10.1155/2022/9636790 . 36339648 PMC9633201

[21] Frederiksen BL, Brown Pde N, Dalton SO, Steding-Jessen M, Osler M. Socioeconomic inequalities in prognostic markers of non-Hodgkin lymphoma: analysis of a national clinical database. European journal of cancer (Oxford, England: 1990). 2011;47(6):910–7. Epub 2010/12/15. doi: 10.1016/j.ejca.2010.11.014 . 21145729

[22] Frederiksen BL, Dalton SO, Osler M, Steding-Jessen M, de Nully Brown P. Socioeconomic position, treatment, and survival of non-Hodgkin lymphoma in Denmark—a nationwide study. British journal of cancer. 2012;106(5):988–95. Epub 2012/02/09. doi: 10.1038/bjc.2012.3 . 22315055 PMC3305955

[23] Kahn JM, Kelly KM, Pei Q, Bush R, Friedman DL, Keller FG, et al. Survival by Race and Ethnicity in Pediatric and Adolescent Patients With Hodgkin Lymphoma: A Children’s Oncology Group Study. Journal of clinical oncology: official journal of the American Society of Clinical Oncology. 2019;37(32):3009–17. Epub 2019/09/21. doi: 10.1200/JCO.19.00812 . 31539308 PMC6839907

[24] Abodunrin FO, Akinyemi OA, Ojo AS, Elleissy Nasef K, Haupt T, Oduwole A, et al. Racial Disparities in Survival Among Non-Hodgkin Lymphoma Patients: An Analysis of the SEER Database (2007–2015). Cureus. 2022;14(6):e25867. Epub 2022/07/16. doi: 10.7759/cureus.25867 . 35836466 PMC9275381

[25] Li Y, Wang Y, Wang Z, Yi D, Ma S. Racial differences in three major NHL subtypes: descriptive epidemiology. Cancer epidemiology. 2015;39(1):8–13. Epub 2015/01/07. doi: 10.1016/j.canep.2014.12.001 . 25560974 PMC4323749

[26] Rossi C, Jégu J, Mounier M, Dandoit M, Colonna M, Daubisse-Marliac L, et al. Risk assessment of second primary cancer according to histological subtype of non-Hodgkin lymphoma. Leukemia & lymphoma. 2015;56(10):2876–82. Epub 2015/02/03. doi: 10.3109/10428194.2015.1007505 . 25641432

[27] Monnereau A, Troussard X, Belot A, Guizard AV, Woronoff AS, Bara S, et al. Unbiased estimates of long-term net survival of hematological malignancy patients detailed by major subtypes in France. International journal of cancer. 2013;132(10):2378–87. Epub 2012/10/05. doi: 10.1002/ijc.27889 . 23034773

[28] Pavlovsky M, Cubero D, Agreda-Vásquez GP, Enrico A, Mela-Osorio MJ, San Sebastián JA, et al. Clinical Outcomes of Patients With B-Cell Non-Hodgkin Lymphoma in Real-World Settings: Findings From the Hemato-Oncology Latin America Observational Registry Study. JCO global oncology. 2022;8:e2100265. Epub 2022/04/30. doi: 10.1200/GO.21.00265 35486884 PMC9088238

[29] Armitage JO, Gascoyne RD, Lunning MA, Cavalli F. Non-Hodgkin lymphoma. Lancet (London, England). 2017;390(10091):298–310. Epub 2017/02/06. doi: 10.1016/S0140-6736(16)32407-2 . 28153383

[30] Liu X, Cao D, Liu H, Ke D, Ke X, Xu X. Clinical Features Analysis and Survival Nomogram of Primary Small Intestinal Diffuse Large B-Cell Lymphoma. Cancer management and research. 2022;14:2639–48. Epub 2022/09/13. doi: 10.2147/CMAR.S369086 . 36090469 PMC9462437

[31] Pugh TJ, Ballonoff A, Rusthoven KE, McCammon R, Kavanagh B, Newman F, et al. Cardiac mortality in patients with stage I and II diffuse large B-cell lymphoma treated with and without radiation: a surveillance, epidemiology, and end-results analysis. International journal of radiation oncology, biology, physics. 2010;76(3):845–9. Epub 2009/06/12. doi: 10.1016/j.ijrobp.2009.02.045 . 19515509

[32] Yin X, You L, Hu X. Role of Radiation Therapy in Mortality among Adolescents and Young Adults with Lymphoma: Differences According to Cause of Death. Cancers. 2022;14(20). Epub 2022/10/28. doi: 10.3390/cancers14205067 . 36291852 PMC9599966

[33] Habash M, Bohorquez LC, Kyriakou E, Kron T, Martin OA, Blyth BJ. Clinical and Functional Assays of Radiosensitivity and Radiation-Induced Second Cancer. Cancers. 2017;9(11). Epub 2017/10/28. doi: 10.3390/cancers9110147 . 29077012 PMC5704165

[34] Chung CS, Liao LJ, Wu CY, Lo WC, Hsieh CH, Lee TH, et al. Endoscopic Screening for Second Primary Tumors of the Esophagus Among Head and Neck Cancer Patients. Frontiers in oncology. 2022;12:906125. Epub 2022/06/25. doi: 10.3389/fonc.2022.906125 . 35747824 PMC9209650

